# Accurate, Efficient and User-Friendly Mutation Calling and Sample Identification for TILLING Experiments

**DOI:** 10.3389/fgene.2021.624513

**Published:** 2021-02-03

**Authors:** Juanita Gil, Juan Sebastian Andrade-Martínez, Jorge Duitama

**Affiliations:** ^1^Systems and Computing Engineering Department, Universidad de Los Andes, Bogotá, Colombia; ^2^Research Group on Computational Biology and Microbial Ecology, Department of Biological Sciences, Universidad de Los Andes, Bogotá, Colombia; ^3^Max Planck Tandem Group in Computational Biology, Universidad de Los Andes, Bogotá, Colombia

**Keywords:** software, mutagenesis, functional genomics, TILLING, variants detection

## Abstract

TILLING (Targeting Induced Local Lesions IN Genomes) is a powerful reverse genetics method in plant functional genomics and breeding to identify mutagenized individuals with improved behavior for a trait of interest. Pooled high throughput sequencing (HTS) of the targeted genes allows efficient identification and sample assignment of variants within genes of interest in hundreds of individuals. Although TILLING has been used successfully in different crops and even applied to natural populations, one of the main issues for a successful TILLING experiment is that most currently available bioinformatics tools for variant detection are not designed to identify mutations with low frequencies in pooled samples or to perform sample identification from variants identified in overlapping pools. Our research group maintains the Next Generation Sequencing Experience Platform (NGSEP), an open source solution for analysis of HTS data. In this manuscript, we present three novel components within NGSEP to facilitate the design and analysis of TILLING experiments: a pooled variants detector, a sample identifier from variants detected in overlapping pools and a simulator of TILLING experiments. A new implementation of the NGSEP calling model for variant detection allows accurate detection of low frequency mutations within pools. The samples identifier implements the process to triangulate the mutations called within overlapping pools in order to assign mutations to single individuals whenever possible. Finally, we developed a complete simulator of TILLING experiments to enable benchmarking of different tools and to facilitate the design of experimental alternatives varying the number of pools and individuals per pool. Simulation experiments based on genes from the common bean genome indicate that NGSEP provides similar accuracy and better efficiency than other tools to perform pooled variants detection. To the best of our knowledge, NGSEP is currently the only tool that generates individual assignments of the mutations discovered from the pooled data. We expect that this development will be of great use for different groups implementing TILLING as an alternative for plant breeding and even to research groups performing pooled sequencing for other applications.

## Introduction

Targeting Induced Local Lesions in Genomes (TILLING) is a powerful reverse genetics method used in plant sciences which allows the identification of point mutations or SNPs, introduced randomly throughout the whole genome by chemical mutagenesis (Missirian et al., 2011). In brief, TILLING consists of mutagenesis, DNA extraction and pooling of several individuals of a population, PCR amplification of regions of interest, and high-throughput mutation discovery in target genes (McCallum et al., 2000). Despite newer technologies being available for targeted modification of genes such as CRISPR-Cas, TILLING remains a useful and effective functional genomics tool for studying genes responsible for desired phenotypes because large populations can be screened for mutations before bringing plants to the field, thus reducing phenotyping costs, and it generates genome-wide mutations allowing to target in multiple genes at the same time (Irshad et al., 2020). With the advance in high-throughput sequencing technologies and their current lower costs, TILLING by Sequencing proves to be the best choice for the identification of mutations and the corresponding mutant individuals in pooled samples, and for linking the identified base pair changes with their impact on specific traits (Tsai et al., 2011).

The application of bioinformatic tools contributes to virtually all elements of the TILLING pipeline, including identification of the genes in the species of interest, amplicon design, and analysis of the effect of produced mutations in protein products (Kurowska et al., 2011). The biggest bioinformatic challenge in TILLING is variant calling in multidimensional experiments. In essence, an efficient pipeline for detection must not only call variants in each pool but triangulate the outputs per pool to identify true variants and determine the individual carrying each mutation based on the specific pooling design (Missirian et al., 2011). Moreover, mutations produced through TILLING are rare within the population. Hence, special efforts must be taken to distinguish true variants from noise (Missirian et al., 2011). While some of the available tools for variant calling are able to detect variants in pooled samples (Huang et al., 2015), they are not designed toward the posterior triangulation of the variants detected from each individual pool. Moreover, most tools require high coverages and high sequencing qualities to achieve good accuracy (Missirian et al., 2011). Accuracy and efficiency vary amply between software tools (Huang et al., 2015). As of today, the only available tool specifically designed for variant calling in TILLING experiments is CAMBa, which employs Bayesian statistics for yielding the most probable mutations in a TILLING experiment per individual (Missirian et al., 2011).

Since the advent of Next-Generation Sequencing (NGS), it has been proposed that TILLING procedures could eventually be carried out totally *in silico* (Wang et al., 2012; Chen et al., 2014). As mentioned above, tools have been developed in the past for *in silico* identification of candidate genes, such as CODDLE (Slota et al., 2017), for analysis of the effects of putative or detected mutations in TILLING populations such as PARSESNP (Taylor and Greene, 2003), SAS (Milburn et al., 1998), or SOPMA (Geourjon and Deleage, 1995), and for variant detection, such as CAMBa and our own implementation. Nonetheless, to the best of our knowledge there is no tool available for simulation of NGS pool-sequencing in the context of multidimensional TILLING experiments. This tool would be critical both for potential fully *in silico* TILLING experiments, as well as for guiding the design of *in vivo* procedures.

The development of new bioinformatic tools to increase the precision of TILLING experiments is crucial, especially when considering TILLING branching applications. Moreover, pooled sequencing for variant discovery is used in other protocols related to crop breeding and even in distant fields such as the study of rare human genetic diseases. Pooled sequencing followed by the identification of *de novo* variants has facilitated the typing of a larger number of donors for stem cell transplants at the same time, increasing the chance of finding a good match for recipient patients (Lange et al., 2014) and can also improve diagnostics rates of genetic disorders by increasing the number of probands tested at a time at a reduced cost (Dashnow et al., 2019). In the context of plant breeding, introducing natural or artificial allelic diversity in crops is widely used to develop new varieties with improved traits that meet the current global demands for food production. Some examples are kernel hardness in wheat (Ma et al., 2017), drought tolerance (Yu et al., 2012), and starch quality (Raja et al., 2017) in rice, seed weight in chickpea (Bajaj et al., 2016), and starch biosynthesis and herbicide tolerance in cassava (Duitama et al., 2017).

We have developed, through the Next Generation Sequencing Experience Platform (NGSEP), two new functionalities for TILLING analyses: a TILLING experiment simulator and a TILLING detector. The simulator is able to generate pool reads derived from any set of genomic sequences, creating an *in silico* population for the experiment with associated variants assigned to specific individuals. The detector leverages NGSEP variant detection to first call variants per pool, which are then triangulated to perform identification of the individuals associated with the discovered mutations.

## Results

### Novel Functionalities for Simulation and Read Analysis in TILLING Experiments

**FIGURE 1:**
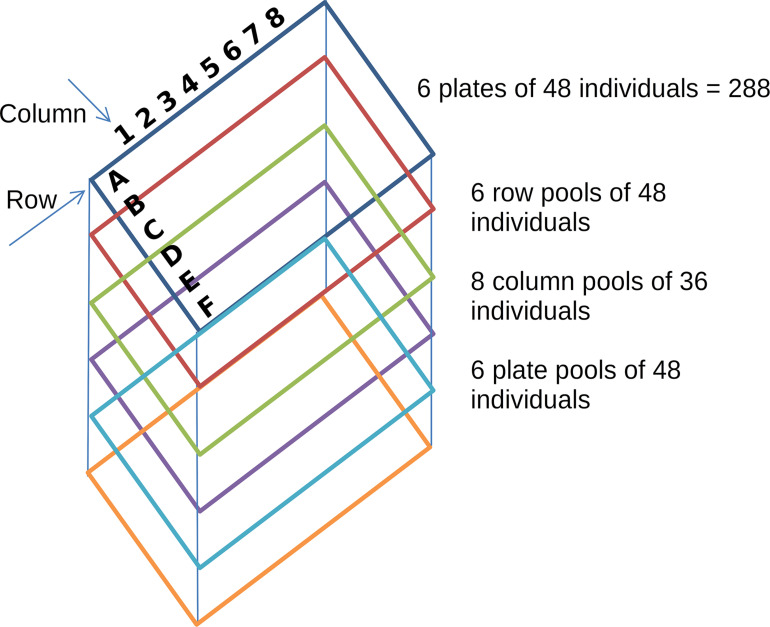
Pooling strategy in a simulated TILLING experiment. Each colored rectangle represents a plate of 6 × 8 wells with 48 individuals; rows are represented by the letters A to F and columns by the numbers 1 to 8. The tridimensional pooling strategy results in 20 pools. Row pools consist of individuals of each single row of each plate (48 individuals). Column pools consist of individuals of each single column of each plate (36 individuals) and plate pools consist of the individuals of each plate.

In a TILLING experiment, a mutagenic agent is used to treat the seeds and induce random mutations across the entire genome of a particular organism. One of the most commonly used agents is ethyl methanesulfonate (EMS), which induces 2 to 10 mutations/Mb of diploid DNA (Henry et al., 2014). Mutagenized TILLING populations are analyzed for the identification of the mutations generated across the individuals of the population. If sequencing occurs after one round of selfing (usually called generation M2), about half of the mutations are heterozygous in the population. Although it is technically possible to sequence independently and call variants on each individual of the population, this procedure is not cost effective given that most of the individuals will not carry interesting mutations and promising individuals usually go over further rounds of selfing to stabilize the mutation and its potential phenotypic effect. Hence, the TILLING by sequencing design suggests a tridimensional pooled strategy in which each individual is included in a unique combination of three different pools, one per dimension (Figure 1). Pools are then sequenced and mutations are identified in the pools. Taking into account the mutation rate, it is very unlikely that two individuals carry exactly the same mutation. Thus, individual assignments can be performed looking for mutations consistently called in three pools of different dimensions. This design allows to perform mutation detection and individual assignment for hundreds of individuals sequencing only the sum of the pools generated for each dimension.

One of the main aspects to take into account for a TILLING experiment is the design of the number of pools to include in each dimension and the number of individuals per pool. To provide a tool to explore *in silico* the behavior of a TILLING experiment in different scenarios, we developed a simulator of TILLING experiments based on a set of target regions from a reference genome, a given population size, a mutation rate, and a design of overlapping pools. Based on this information, the simulator follows *in silico* the steps of mutagenesis, sample pooling, and sequencing. Regions to be amplified for each individual in the population were created first as an exact copy of the reference and then mutations were assigned randomly to each individual at a random position and to a random base pair, distinct from the reference. According to the pooling design, each individual was assigned to a row, column and plate pool. Given the sensitivity limitation of the variant calling process of mutations occurring at low frequencies, the smaller the population and the number of samples per pool, the higher the probability of calling true mutations. Therefore, we proposed an experimental design of overlapping amplicons per target gene for a population of 288 individuals. By having pools of maximum 48 individuals (96 haplotypes) we reduced the noise caused by the simulated and expected sequencing errors.

Paired-end high throughput sequencing (HTS) reads were simulated for each pool from the *in silico* mutated amplicon sequences. Mimicking the actual sequencing process and the known error rate patterns of Illumina, a read was generated for each pool selecting a random amplicon within the pool. A forward and a reverse read of a given length were then simulated starting from each end of the selected amplicon. Given a minimum and maximum error rate, the simulator generates substitution errors at random according to a stepwise distribution which starts from the minimum error rate at the 5’ end and ends with the maximum error rate at the 3’ end of the read.

We tested the performance of our simulator by recording the time and memory spent during different simulations varying the number of individuals of the population, dimensions of the pooling design, read lengths, and sequencing depths (Table 1). In all cases, the simulator ran in less than 2 h, and in less than 1 h for all cases of 50X and 10X coverage. In general, time is affected mostly by the number of reads, with simulations of similar coverages running faster with longer read lengths. Memory requirements did not exceed 3 GB in any case. This factor was mainly determined by the size of the simulated population.

**TABLE 1:** Running times (in seconds) and memory requirements (in MB) of three different TILLING experiment simulations ran by the simulator: 8 × 12 row by column plates for 800 individuals, 8 × 8 plates for 800 individuals, and 6 × 8 plates for 288 individuals with a total of 300 mutations within the population.

			Time (s)			Memory (MB)		
Dimension (row × column × plate)	Population size	Read length (bp)	Depth			Depth		
			10X	50X	100X	10X	50X	100X
8 × 12 × 9	800	200	404.04	1933.18	3961.07	2215.70	2215.70	2211.86
		100	638.88	3315.90	6883.64	2215.70	2215.70	2211.84
8 × 8 × 13	800	200	281.81	1283.45	2660.51	2215.70	2215.70	2215.70
		100	498.09	2389.13	4422.11	2215.69	2215.70	2211.84
6 × 8 × 6	288	200	146.75	721.99	1325.39	2139.41	2139.42	2139.46
		100	245.22	1182.25	2509.16	2139.40	2139.42	2139.42

*For each case, coverage of 10X, 50X, and 100X was tested, for 100 and 200 bp read length.*

We also developed modifications of the core algorithm for variants detection available in NGSEP and a new functionality to perform the specific analysis of HTS data required by TILLING experiments. From the algorithmic perspective, the discovery of mutations within each pool is the most challenging part of the analysis because mutations are expected to be carried by one or at most two haplotypes within each pool. Hence, the variants discovery module should be able to separate true variants with allele frequencies of one divided by the number of haplotypes in a pool from sequencing errors. As detailed in the next section, we modified the Bayesian model implemented in NGSEP to identify mutations in these circumstances. Once mutations are identified, and taking into account the pooling strategy, the main outcome of a TILLING experiment should not only be the identification of true mutations but the identification of individuals carrying these mutations. Hence, we developed a module that receives the individual VCF files with variants called within each pool and a text file with the configuration of samples included in each pool, and performs the individual sample genotyping of the mutations (also called triangulation). Taking into account that each sample is included in a unique combination of pools, a variant is assigned to a sample if and only if it is called in all pools in which the sample was included. The triangulation module traverses in parallel the pool VCF files and, for each mutation identified in three pools of different dimensions, queries the pool configuration information to determine which individual is present in the three pools and assigns the mutation to such individual. The output of this process is a VCF file with one column per individual, which in simulation experiments can be directly compared with the VCF gold standard file produced by the simulator.

### Variant Detection and Genotyping in Polyploid Individuals and Pools

We modified the core module of NGSEP (the variants detector) with two related goals: to improve the accuracy of variant calling in polyploid individuals and to allow identification of variants at different allele frequencies in pooled samples. First, sites in which at least one allele different from the reference is observed with a count at least 0.5/*a* were identified, where *a* is the total number of haplotypes in the sample. For a pool of *n* individuals with ploidy *p*, the total number of haplotypes a would correspond to *n*^∗^*p*. For each selected site, the algorithm calculates the conditional probability of the data assuming a homozygous genotype for the allele with the highest read count and the conditional probabilities of the data assuming each possible heterozygous allele dosage for the allele with the second read count, from 1/*a* to 0.5. Both the homozygous genotype and the heterozygous genotypes can be encoded as m copies of a major allele *G*_1_ and *a*–*m* copies of a minor allele *G*_2_, where *m* ≥ 0.5^∗^*a*. A value of *m* = *a* would correspond to a homozygous genotype. Similar to the case of a single diploid individual, given a pileup position of the genome and the set *R* of reads spanning that position, the conditional probability of *R* given the genotype *G* = *G*_1_*^*m*^ G*_2_*^*a*^*^–^*^*m*^* can be calculated as the product of the conditional probability of each read *r* ∈ *R* given *G*. Calling b the base pair of *r* spanning the analyzed position, *e* its error probability and *f* = *m*/*a* the frequency of the major allele, the conditional probability *P*(*b*| *G*) is given by this formula:

$$P\left(b|G_{1}^{m}G_{2}^{a-m}\right)=\left\{\begin{array}{c} 1-e,a=m\wedge b=G_{1}\\ \frac{e}{3},b\neq G_{1}\wedge b\neq G_{2}\\ f\left(1-e\right)+\frac{\left(1-f\right)e}{3},a<m\wedge G_{1}=b\\ \left(1-f\right)\left(1-e\right)+\frac{fe}{3},a<m\wedge G_{2}=b \end{array}\right\}$$

Similar to the case with diploid individuals, a prior probability *P*(*G*) can be calculated from previous knowledge on heterozygosity rate. We set a non-informed prior in our experiments with simulated and real data.

### Comparison of Variant Calling Tools

Comparison of the performance of different variant calling tools was carried out based on the simulated sequences. As observed in the simulations and real data, the sequencing error rate becomes the most critical factor to determine the number of total haplotypes (and by extension samples) that can be included within each pool to be able to separate true mutations from sequencing errors. Given an average sequencing error rate of 0.5% we could achieve good accuracy with up to 64 diploid individuals (128 haplotypes) per pool. Hence, we present here the results of the simulation experiment with 288 individuals arrayed in 6 plates of 6 × 8 rows by column set up (Figure 1).

The results of the variant detection step obtained with our algorithm were compared with the results obtained from other tools frequently used for variant detection, such as GATK haplotype caller (McKenna et al., 2010) and Freebayes (Garrison and Marth, 2012), as well as tools designed to identify low frequency variants like Lofreq (Wilm et al., 2012), or to identify variants in pools like CRISP (Bansal, 2010), and SNVer (Wei et al., 2011). Freebayes was the only tool that did not identify variants in any of the pools of the simulation experiments and was not considered for further comparisons. Given that the output VCF file generated by SNVer is outdated and could not be modified to run the comparisons, this tool was also discarded for comparison purposes. We were unable to run CAMBa (Missirian et al., 2011) by ourselves nor received a response after trying to contact the developers, so we omitted said tool.

**FIGURE 2:**
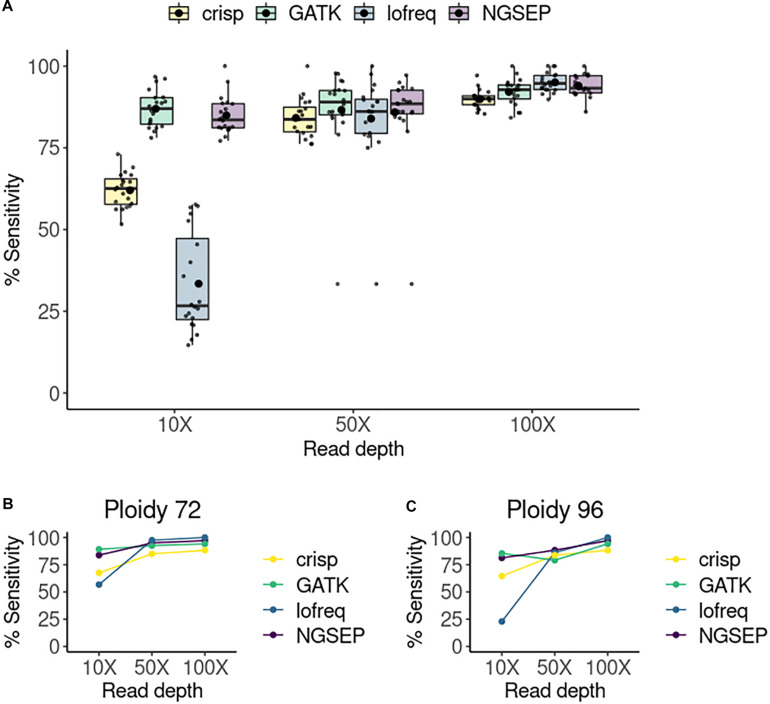
Sensitivity of variant calling per pool. **(A)** Sensitivity of variant detection in a simulated TILLING population comprising 288 individuals sequenced in 20 pools. The small points represent each single pool. The box plots represent the median and first and third quartiles of the sensitivity in all pools. The bigger black point shows the average sensitivity of all pools per tool and read depth. **(B)** Sensitivity of variants detected in a randomly selected pool with 48 individuals (ploidy equal to 96 haplotypes) at three different read depths. **(C)** Sensitivity of variants detected in a randomly selected column pool with 36 individuals (ploidy equal to 72 haplotypes).

We compared the tools in terms of their sensitivity, expressed as the number of true positives divided by the sum of true positive and false negative values. For each of the 20 pools, sensitivity was calculated and compared between the four selected variant callers and for each experiment varying the read depth (Figure 2A). We also tried to calculate specificity but it was 100% in all cases. CRISP consistently showed the lowest sensitivity among the tools and read depths. Lofreq showed improved performance with increasing read depth, showing the best results of all tools at a coverage of 100X, but the worst sensitivity at a coverage of 10X. GATK and NGSEP both showed consistent high sensitivities at all read depths. While GATK shows slightly higher sensitivities than NGSEP at 10X and 50X of coverage, NGSEP performs slightly better than GATK at 100X coverage. We also compared sensitivities in randomly selected pools by varying the total number of haplotypes or ploidy (Figures 2B,C). At low coverage (10X) Lofreq is the worst performing tool regardless of this number. However, it performs two times better calling variants in pools with less haplotypes. This is evident by the sensitivity drop from 57% in the pool with 72 haplotypes (36 individuals) to 23% in the pool with 96 haplotypes (48 individuals). However, this tool outperforms CRISP and GATK in these two particular pools selected for comparison at higher coverages (50X and 100X). Smaller variations in sensitivity were observed in the other three tools when comparing specific pools with two different ploidies at different read depth, with NGSEP and GATK showing the most similar sensitivity values between both samples across the coverage range.

**FIGURE 3:**
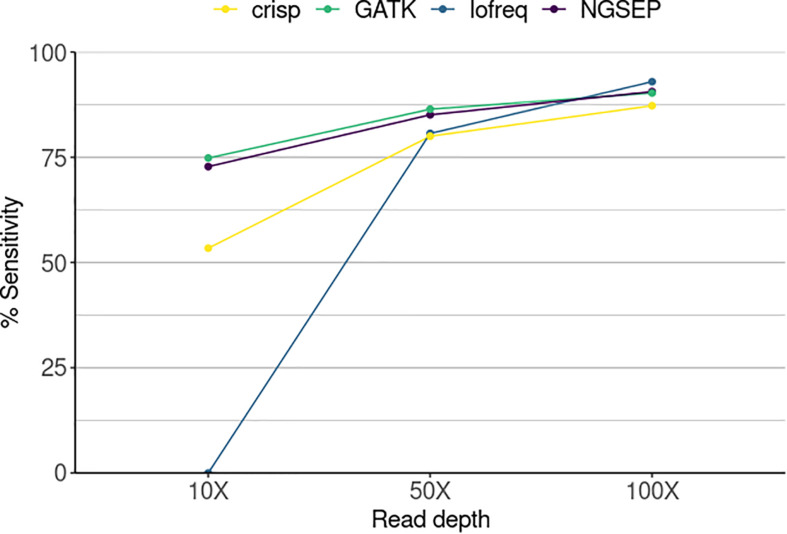
Sensitivity in the identification of mutant individuals in a simulated TILLING population comprising 288 individuals sequenced in 20 pools and three individual experiments varying the sequencing read depth. Sensitivity corresponds here to the number of SNPs detected by each tool divided by the total number of SNPs in a gold standard.

We used our new functionality to identify the individuals carrying mutations in a simulated population and compared the ability of each variant caller to successfully call variants in overlapping pools. Sensitivity was determined as the total number of SNPs identified over the total number of SNPs that should have been detected corresponding to the simulated mutations (Figure 3). The sensitivity of Lofreq was zero at the lowest simulated sequence coverage. This means that although the tool is able to call variants in every pool as shown in Figure 2, those are not found in the three pools that overlap and the SNP cannot be assigned to any individual. Nevertheless, its performance improved with increasing coverage calling between 80 and 93% of the mutations that correspond to one single individual. CRISP showed the poorest performance among the four compared tools at read depths of 50X and 100X. It reached, however, a sensitivity equal to and above 80% at the two highest read depths, respectively. NGSEP and GATK are the best performing tools regardless of read depth. However, with increasing read depth both tools showed higher sensitivities, reaching 90.6 and 90.3% at 100X, respectively. Although sequencing depth improves the sensitivity of the tools, both NGSEP and GATK can detect around 75% of low frequency SNPs in a mutated population and those can be correctly assigned to mutated individuals at a read depth as low as 10X.

**FIGURE 4:**
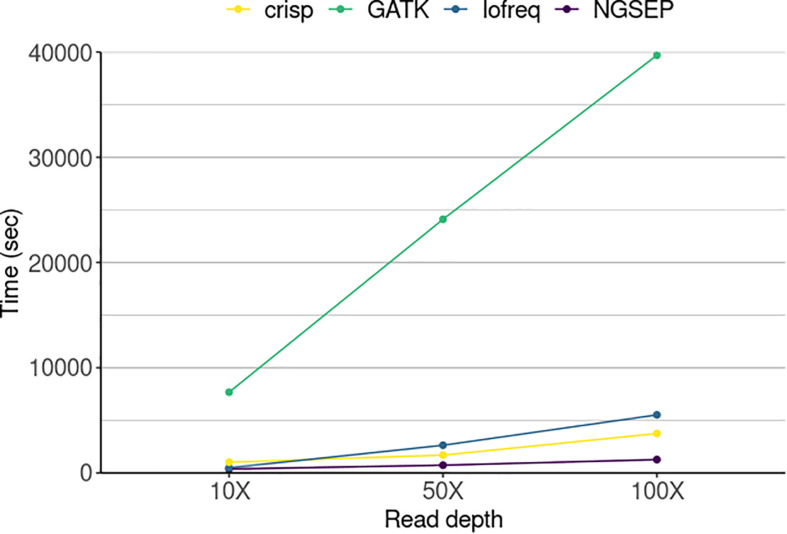
Running time of four variant detector tools for a simulated TILLING population comprising 288 individuals sequenced in 20 pools in three different experiments varying sequencing read depth. The time represents the amount of seconds used by each tool to call variants in 20 pools.

Running times spent by each tool were compared to further assess the performance of the variant calling process (Figure 4). NGSEP was the most efficient tool even at the highest sequencing read depth. GATK was the slowest of all tools taking up to 12 h (∼40,000 s) to call variants in 20 pool samples of a population of 288 individuals, while the other tools required a maximum of 1.5 h to perform the same job. NGSEP showed the most steady time performance over increasing read depths. Variant calling in the simulated experimental setup only took 6.2 min at 10X coverage, 12.3 min at 50X and 21.2 min at 100X when running NGSEP on a laptop.

### Analysis of a Rice TILLING Population

**FIGURE 5:**
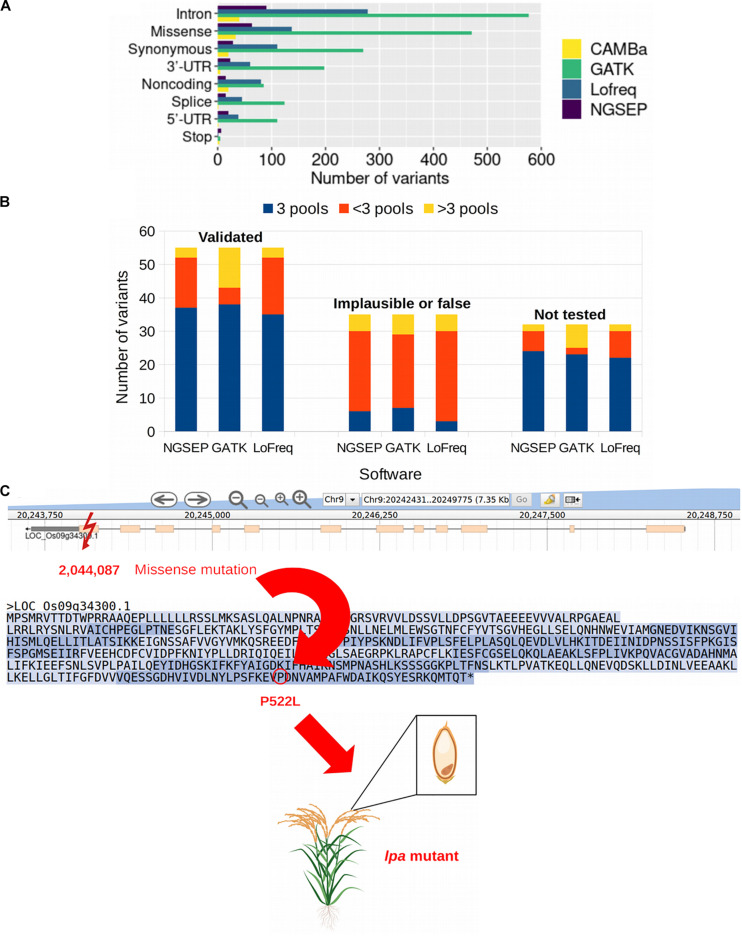
Mutations in a rice TILLING population. **(A)** Number of variants identified in a rice TILLING population comprised by 768 individuals categorized by the type of variant. NGSEP, GATK, and Lofreq were the variant caller used in this study and variants reported by CAMBa were obtained from Tsai et al. (2011). **(B)** Mutations called in pools by three software, NGSEP, GATK, and Lofreq, classified into three categories based on results by Tsai et al. (2011): Validated, which are verified mutations; implausible or false, which are mutations found in non overlapping pools or are false positives; and not tested, which are not verified mutations. Within the category of validated mutations, and possibly also within the not tested category, most of the mutations are expected to be assigned to three pools (blue bars). In the implausible or false category most of the mutations should be found in <3 pools or >3 pools (red and yellow bars, respectively). **(C)** Example of a missense mutation in gene Os09g34300. The mutation at position 2,044,087 on chromosome 9 results in the amino acid substitution from proline to leucine amino acid at position 522 in the protein, leading to reduced phytic acid (lpa) content in the grains of the mutant plants with no negative effect on grain weight or delayed seedling growth (Kim and Tai, 2014).

We used the publicly available data of a sequencing experiment of 44 pools from a rice TILLING population comprising 768 individuals and 32 gene fragments that added up to 42,034 bp. Tsai et al., 2011 reported 122 mutations in overlapping pools detected with the tool CAMBa (Missirian et al., 2011) in this dataset. We identified 262 biallelic SNVs in those pools using NGSEP, 1,852 with GATK, 751 with Lofreq and 0 with CRISP. Despite having an acceptable to good performance on simulated data, none of the SNPs called by CRISP passed the quality filter using the rice population and the VCF files could not be used for the triangulation process in which mutations are assigned to individuals. We calculated exact genomic positions for all SNPs reported by Tsai et al., 2011 to assess if the SNPs detected by NGSEP, GATK, and Lofreq corresponded to the previously reported mutations including the same expected effect on the corresponding gene based on the annotation of the VCF files (Supplementary Table 1). The three variant calling tools used to test the new triangulation function of NGSEP identified more than 122 mutations. NGSEP reported 262 mutations (Supplementary Table 2), GATK 1,852 (Supplementary Table 3), and Lofreq 751 (Supplementary Table 4). We compared the results according to the number of variants detected by each tool (NGSEP, GATK, and Lofreq in this study and CAMBa in the previous study) and the type of variant (or predicted effect) on the sequenced gene (Figure 5A). The most common type of variant was intronic variants with 90 mutations being identified by NGSEP, 577 by GATK, 279 by Lofreq, and 40 by CAMBa. Missense and synonymous variants were the second and third most common type of variants identified by all tools. The least frequent type of variant was mutations leading to a stop codon. Regarding the type of SNVs identified in the mutated rice samples, the most common were *G* to *A* and *C* to *T* transitions according to the results obtained with NGSEP (32.4%) and CAMBa (66.9%). Conversely, AT to GC transitions were the most frequent mutation type for GATK (89.15%) and Lofreq (65.9%). These transitions were found only in 27.9% of the mutations reported by NGSEP and 19% of the mutations reported by CAMBa. In accordance with Tsai et al., 2011 *G* to *C* or *C* to *G* transversions were the least common (<5.2% of all mutations for all tools). Overall, there were more transitions than transversions (Supplementary Table 5). Although NGSEP reported the highest percentage of transversions (35.11%), looking at the number of pools where these transversions are called, we found that transversions were called in more pools than transitions (Supplementary Table 2). Applying a filter keeping only variants called in at most six pools, the percentage of GC > AT transitions increased to 47.13% for NGSEP and the percentage of transversions reduced to 16.09%. GATK and Lofreq also reduced the percentage of transversions after this filter but preserved the excess of AT > GC transitions, compared to the results originally reported using CAMBa.

In the previous study conducted by Tsai et al., 2011, the mutations were categorized into homozygous, heterozygous, implausible or false based on validation experiments, and not tested for mutations that were not validated. We used these categories to analyze how many of the mutations were found in exactly three, more than three or less than three pools within each category using NGSEP, GATK, and Lofreq (Figure 5B). We found that within the validated mutations more than 67% of them were assigned to exactly three pools by each tool, which is the expectation for tridimensional pooling strategies. In contrast, within the category containing implausible or false positive mutations, less than 20% of the mutations were assigned to exactly three pools, and found principally in less than three pools (red bars in Figure 5B). Within the not tested category, 75, 71.9, and 68.8% of the mutations were assigned to exactly three pools for NGSEP, GATK and Lofreq, respectively, and could potentially be true mutations in the population. Considering the number of variants assigned to a number of pools different than three, GATK reported 12 validated variants in more than three pools, whereas this number was only three for NGSEP and Lofreq. Conversely, validated variants called in less than 3 pools were only 5 for GATK, whereas this number was 15 and 17 for NGSEP and Lofreq, respectively. This behavior is consistent for the non tested variants and reflects that GATK predicted a much larger overall number of mutations.

To further validate the performance of the new functionalities in NGSEP using real data, we selected two of the genes for which mutations have also been reported elsewhere and their effect has been described. The inositol kinase-like gene Os09g34300 and the multidrug resistance-associated gene Os03g04920 are both involved in the reduction of phytic acid (myo-inositol 1,2,3,4,5,6-hexakisphosphate) in rice seeds. Phytic acid is considered an antinutrient because humans and other non-ruminants are unable to efficiently digest it and it prevents the absorption of important micronutrients in their intestines (Perera et al., 2019). Four novel mutations obtained by TILLING were reported by Kim and Tai (2014) within these two genes, obtaining four low phytic acid (lpa) mutants, two of which were similar to wild-type plants in seed weight, germination, and seedling growth. One missense mutation in the last exon of the gene Os09g34300 leading to the amino acid change P522L was identified by NGSEP, GATK, and Lofreq as well. This mutation was also reported and validated in (Tsai et al., 2011) and confirmed as a lpa mutant by (Kim and Tai, 2014) in the laboratory, being a promising line for breeding in rice programs aiming at developing varieties with improved nutritional quality. Within this same gene, an intronic and a splice variant not tested in the study of (Tsai et al., 2011) were also identified using the three variant callers. The position and effect of the mutation in the inositol kinase-like gene are schematically shown in Figure 5C. Both of the reported mutations by (Kim and Tai, 2014) within gene Os03g04920 that led to a lpa phenotype and were also included in the list of mutations by (Tsai et al., 2011) were identified by NGSEP, GATK and Lofreq as well. Furthermore, from the seven validated mutations within this gene, NGSEP, GATK, and LoFreq called five of them in exactly three pools. From the six non tested variants, NGSEP and GATK called five and Lofreq called four in exactly three pools.

## Discussion

Mutagenesis is a widely used experimental technique in functional genomics because it allows to generate genetic variability not present in natural populations in an unbiased manner. The TILLING experimental setup reduces the cost needed to identify mutations in candidate genes across populations developed through mutagenesis and to perform the identification of mutated individuals (Kurowska et al., 2011). Functional effects of identified mutations can then be further investigated using protein modeling or even through more directed approaches such as CRISPR, besides the observation of the expected phenotype. Although recent technologies have been developed for targeted genome editing, including CRISPR, the resulting organism is usually considered a genetically modified organism (GMO) presenting an important problem and limitation in plant breeding for the release of improved varieties into the markets of countries with strict regulations about GMOs. Mutagenesis, however, has been considered a safe method to rapidly induce genetic variation and develop improved varieties, that are not regulated by the GMO legislations (Holme et al., 2019). Moreover, since TILLING samples come from a population whose individuals (or their seeds) are readily available for the researcher (Wang et al., 2012), individual identification is particularly useful because it allows to perform validation of the potential phenotype differences generated by the identified mutations in the associated individuals, providing valuable information for future plant breeding.

In this work, we presented new functionalities of NGSEP to facilitate the data analysis steps required to obtain the expected information from a TILLING experiment. First, we developed an improved model to perform accurate variant identification in pooled samples, which is useful for different applications of HTS. Either in mutagenized or in natural populations, variants could be quickly identified by bulk sequencing of large numbers of individuals to avoid the costs of sample by sample barcoding and library preparation. Germplasm banks are using pooled sequencing to validate genetic stability of accessions avoiding the cost of individual sequencing of potential clones (see for example Rubinstein et al., 2019). Moreover, the same underlying model to perform pooled genotyping might be useful to perform individual genotyping in species with high ploidy such as sugar cane, where genotyping by sequencing is preferred over SNP arrays for variant detection (Manimekalai et al., 2020). We show through simulation experiments that our model has comparative accuracy and better efficiency compared to other solutions. NGSEP showed consistently high sensitivities (above 80%) across varying read depths (10X, 50X, and 100X) in variants called in individual pools in simulated data. GATK also showed good sensitivities in pool variant calling at different read depths. Conversely, Lofreq and CRISP only showed good performances (sensitivity > 75%) at higher read depths (50X and 100X). Moreover, we show how different tools for regular variant identification should be adapted to increase sensitivity in pooled data. This is very important for mutagenesis experiments because identification of mutations present in only one haplotype of the pool is the most challenging case of pooled variant identification, with different variant callers achieving different performances depending on sequencing depth (Huang et al., 2015). Hence, researchers struggle trying to adapt individual genotyping tools to experimental setups including pooled sequencing.

Particularly for TILLING, we developed a functionality to perform individual assignment of variants from the information of individuals included in each pool. To the best of our knowledge, NGSEP is currently the only open source software able to perform this step of the analysis process. Moreover, both the variant identification and the individual assignment can be executed from the graphical interface of NGSEP. Finally, we also built a functionality to perform simulations of TILLING experiments. Besides being useful to perform benchmarking of current and future analysis pipelines developed by different research groups, the simulator can also be used to validate the effectiveness of different pool configurations to achieve the goals of the experiment, saving time and money in *in vivo* analyses. This is particularly important given that preparation of populations for TILLING analysis is a long and costly process (Wang et al., 2012), so *in silico* experiments can help to make large-scale TILLING procedures more cost-effective.

We analyzed a large rice mutant population for high throughput mutation identification using the approach of TILLING by sequencing and a tridimensional pooling strategy. Using the publicly available sequences from a previous study by Tsai et al. (2011) we compared the performance of four variant calling tools, NGSEP, GATK, Lofreq, and CRISP. CRISP was discarded from the comparisons using real data because the SNPs called in pools did not pass the quality filter. In the previous study, the authors reported 122 mutations in the population using the tool CAMBa, developed by the same group. After filtering by number of pools, with NGSEP we identified 87 mutations, which was the second closest result compared to the previous report. On the other hand, GATK and Lofreq identified 569 and 127 mutations, respectively. Considering the small fragment of the genome that was targeted during the TILLING experiment, the number of mutations reported by GATK would represent an unexpectedly high mutation rate for the mutagenesis experiment (Till et al., 2007). Moreover, both GATK and Lofreq reported an excess of AT > GC transitions which was not observed in the results reported by NGSEP and CAMBa. The analyzed rice population was treated by EMS mutagenesis, which has a G-alkylating action favoring primarily GC to AT base pair transitions. This corresponds to the result obtained with NGSEP and the previous report from CAMBa. The raw output of NGSEP showed a large percentage of transversions (39.7%), while the other tools reported less than 22%. However, most of the transversions were easy to filter out because they are found in a large number of pools, which is not expected in a TILLING experiment due to the low probability of finding a given mutation in more than one individual. Possible explanations for these variants are natural variation between the parent and the reference genome or systematic errors producing consistent false positive calls among pools.

From the set of validated mutations of the study carried out by Tsai et al. (2011), NGSEP, GATK, and Lofreq detected 67.3, 69.1, and 63.6% of them. Assuming a 100% success rate in the verification experiment, the performance of these tools in terms of sensitivity is lower using real data than those obtained using the simulated data of an artificial mutant population. Nevertheless, considering that the tools called mutations in three overlapping pools in less than 20% of the cases of implausible or false positive variants (again assuming this classification is 100% accurate) and that up to 75% of the not tested mutations in the study by (Tsai et al., 2011) were called and identified in three overlapping pools, the three tools show promising results for the analysis of large TILLING populations. From these three tools, NGSEP is the only one that offers the functionality of identifying mutations in overlapping pools and assigning mutations to the corresponding mutant individual in the population. Regarding computational efficiency, NGSEP was the most efficient tool, calling variants in all 44 pools of the rice TILLING population comprising 768 individuals with average sequencing coverage per pool ranging from 300X to 31,500X with the computational resources of a laptop and in less than 2.5 h.

With an ever growing population, the demand for food is increasing around the world. However, to increase crop productivity in a timely manner as required by the necessity of meeting the current global demands, it is critical to explore all possible alternatives to develop plants that are higher yielding and more resilient to climatic changes and their associated problems such as the raise of different pests and diseases or variable abiotic stresses such as drought, higher temperatures, and flooding, among others. We expect that the new developments presented in this manuscript will be useful for researchers implementing TILLING and other experimental techniques for functional genomics and breeding.

## Methods

### Software Development and Implementation Details

We implemented the TILLING simulator and the functionality to perform individual assignments of discovered variants (also called triangulation process) as new functionalities of NGSEP. This allows to have these new functionalities integrated in the same software solution implementing the variant discovery step. Hence, the software is implemented in Java, following an object oriented design. The algorithm to perform variant discovery in pools was implemented within the general functionalities of NGSEP to perform single sample and multisample variants discovery. The new developed algorithm is activated when the number of haplotypes in the pool is provided in the “ploidy” option of these two functionalities. The three functionalities, namely simulation, variant calling, and triangulation, can be executed either from the command line or from the graphical interface of NGSEP v4, built in JavaFX (manuscript in preparation). NGSEP is distributed as an open source software solution available in http://ngsep.sf.net.

In the simulation process, given the pool dimensions selected by the user, the simulator will assign pools to each individual distributing the samples in the plates (wells) from left to right and from the top to the bottom, starting from the first plate to the last one. Depending on the number of individuals and plate size, some pools might contain less individuals than those of a full plate. For example, if a 12 by 8 plate configuration is selected, and the number of individuals is set to 100, then the plate pool for the second plate will only have four individuals, since the remaining 96 are located in the first plate. This implies that different pools will have different numbers of samples and, therefore, different numbers of total haplotypes. Although large populations can be analyzed, pools containing more than 96 individuals should be avoided.

To simulate errors for each read, the range between the minimum and the maximum rates is split into n intervals, where n is the read length. For the nth base in each read, a random decimal within the nth interval is selected and used as the error probability. This number is converted to a quality score for the fastq file. With the decimal selected, a random integer between 0 and 1 is generated, and if it is smaller than the latter, a random base different from the correct one is placed in that position to simulate an error.

The simulator produces a series of files with a given prefix. The first one is a VCF file with the simulated mutations generated for each individual. This file serves as a gold-standard for benchmark experiments. The second is a csv file that indicates which row, column and plate pool is associated with each individual in the population. Two fastq files are generated for each pool according to the current standard for paired-end sequencing. Read ids include the associated individual from which it was obtained, the pool number, and a unique identifier.

### Data Sets

#### Simulated TILLING Dataset

We tested the newly added functions to NGSEP using two datasets. The first one was derived from the simulator: we selected eight genes in common bean (*Phaseolus vulgaris L.*) that are considered to be important for agronomic traits in this crop such as seed color, resistance to herbicides and tolerance to drought, among others. For each gene, primers were designed using the online tool primer3^1^ to generate amplicons that ranged from 279 to 621 bp and covered all exons in each gene when possible. Overlapping amplicons were designed to improve coverage of the target regions. The simulation was run for a population of 288 diploid individuals in 6 × 8 plates, with a read length of 100 bp and coverage of either 10X, 50X, and 100X. The pool design and population size leads to a total of 48 individuals per row and plate pool and 36 individuals for the column pools (Figure 1).

Time and memory benchmarking of the simulator were performed by running other two simulations, along with the one mentioned above. For both of the other simulations we considered a population of 800 individuals with 300 mutations, one with an 8 × 8 plate design and another with an 8 × 12 design. Simulations were run on a Desktop Computer with an Intel Core i7-6700 CPU @ 3.40 GHz, 16 GB of memory and Windows 10 operating system. Times and memory usage were recorded in Java.

#### Rice Dataset

The raw sequencing reads of a TILLING experiment described in Tsai et al. (2011) were downloaded from the SRA NCBI database (BioSample: SAMN00715843) and mapped to the rice reference genome *Oryza sativa* v7.0. This experiment included 768 individuals sequenced in 44 pools with maximum 64 individuals per pool.

### Read Mapping and Variant Calling

All reads in fastq format were mapped to the respective reference genome of the corresponding organism, *Phaseolus vulgaris* for the simulated data and *Oryza sativa* for public data, using NGSEP option ReadsAligner with following parameters modified from the default settings: -k 20 and -m 1. Obtained bam files were then sorted by coordinate using Picard 2.23.0^2^. Alignment rates of 100% were obtained for all mapped reads.

For benchmarking TILLING variant calling and triangulation through NGSEP, we tested a total of 5 additional variant callers in the same datasets: CRISP (Bansal, 2010), Lofreq (Wilm et al., 2012), Freebayes (Garrison and Marth, 2012), GATK (McKenna et al., 2010^3^), and SNVer (Wei et al., 2011). To the best of our knowledge, the only tool capable of identifying mutant individuals in overlapping pools is CAMBa (Missirian et al., 2011). We were unable either to run CAMBa by ourselves nor received a response after trying to contact the developers, so we omitted said tool.

For a fair comparison between tools we adjusted different parameters for variant calling as follows: CRISP, —use duplicates was set to 1 and —qvoffset to 33. For LoFreq, we used the —no-default-filter option and set -m to 20. For Freebayes, the —pooled-discrete option was used and —min-mapping-quality was set to 20. For GATK, we used the HaplotypeCaller algorithm with option —heterozygosity equal to 0.5 and option —max-reads-per-alignment-start set to 0. Finally, we ran SNVerPool with default parameters. SNVer and CRISP allowed us to specify and include the ploidy of each pool (either 72 or 96 depending on the specific pool) in the input file containing the names or paths to the bam files of each pool. Freebayes and GATK were run independently for each pool setting the ploidy to 72 for all column pools and to 96 for the row and plate pools. Lofreq is designed to call low frequency variants and does not have a ploidy option. Finally, for NGSEP, we ran the SingleSampleVariantsDetector functionality with options -h equal to 0.5, -maxAlnsPerStartPos set to 0, -maxBaseQS set to 100 (for real data this option was set to 30), and -psp. Ploidy was adjusted based on the specific pool as explained for the tools above. The commands and parameters are provided in Supplementary File 1.

### Comparison of Variant Callers Performance

Performance of four of the variant callers was determined in terms of the time spent to call variants in all 20 pools of an artificial mutant population comprising 288 individuals. All tools were tested on a laptop with 4 GB memory, Intel Core i5-7200U CPU @ 2.50 GHz × 4 and Ubuntu 20.04.1 LTS as operating system.

Accuracy of four of the variant callers was determined in terms of the number of variants correctly called in each pool. First, the pool gold standard vcf was generated using the class TillingIndividualVCF2PoolVCF in NGSEP. Then, the function VCFFilter was used to generate the gold standard vcfs per pool using the options -saf to provide the pool ID to be filtered out each time and -fi to filter out sites in which only one allele was observed. Finally, the function VCFGoldStandardComparator was used to compare the vcfs obtained from the variant calling step with the gold standard for the same pool. The output of this comparison is a text file that includes the number of true positives (TP), false negatives (FN), and false positives (FP) detected after variant calling, among others. These values were used to calculate the sensitivity of each tool expressed as TP/(TP+FN).

### Identification of Individuals Carrying the Mutations

With the exception of SNVer and CRISP, all variant callers generate a single VCF per pool. The VCFs from SNVer and Crisp include all the samples in one single file. The VCF file generated by lofreq is outdated and does not provide the genotypes per sample. We designed custom scripts to fix the output files of lofreq and crisp. Once fixed, the output VCFs obtained from CRISP were filtered using the option VCFFilter from NGSEP to generate individual VCFs per pool from the population VCF. The parameters used were -saf to provide the pool ID to be filtered out from the original VCF and -fir to remove sites in which only the reference allele was observed. The output VCF obtained from SNVer does not provide information about the observed allele frequencies per sample and could not be fixed to generate a file that could be filtered with NGSEP to generate the individual files. Once we had the VCF files per pool from each tool, we triangulated the output of each caller using the TillingPoolsIndividualGenotyper functionality. Briefly, the genotyper triangulates the calls of all possible trios of pools (overlapping pools) and then assigns mutations to each individual using the information of the row, column and plate pool to which every member of the population is associated, which was obtained from the simulation process. The output VCF was then compared to the gold standard VCF that contains the true mutations in each individual of the population using the option VCFComparator in NGSEP. Sensitivity was determined as the number of SNPs identified by each tool over the total number of SNPs in the individual gold standard VCF.

### Analysis of TILLING Data

Variant calling in the rice TILLING population was performed using NGSEP SingleSampleVariantsDetector, the GATK HaplotypeCaller, Lofreq and CRISP modifying the same parameters as for the simulated data. Ploidy was adjusted according to the pooling strategy described in Tsai et al. (2011) for row, column and dimension (plate) pools varying from 96 to 128. Single vcfs per pool were subjected to the triangulation process using the functionality TillingPoolsIndividualGenotyper providing a pools descriptor file that we generated based on the size of the population (768 individuals) and sampling strategy used in their study. The final vcf was annotated using the function VCFAnnotate and filtered with the function VCFFilter in NGSEP to keep only biallelic SNVs. Summary statistics were calculated using the function VCFSummaryStats of NGSEP.

## Data Availability Statement

Publicly available datasets were analyzed in this study. This data can be found here: Data from the rice population analyzed in this study is available at the sequence read archive (SRA) of NCBI with biosample accession number SAMN00715843.

## Author Contributions

JG, JA-M, and JD conceived the study. JA-M and JD developed the software components, JG and JA-M performed the simulation and comparison experiments and analyzed the rice population. All authors contributed to the manuscript and approved its final version.

## Conflict of Interest

The authors declare that the research was conducted in the absence of any commercial or financial relationships that could be construed as a potential conflict of interest.

## Footnotes

1 https://bioinfo.ut.ee/primer3/

2 http://broadinstitute.github.io/picard/

3 https://www.biorxiv.org/content/10.1101/201178v3

## Supplementary Material

Supplementary Table 1 Correspondance between variants detected by NGSEP, GATK, and Lofreq in the rice TILLING population and those reported by Tsai et al., 2011.

Supplementary Table 2 Raw variants detected by NGSEP in a rice TILLING population.

Supplementary Table 3 Raw variants detected by GATK in a rice TILLING population.

Supplementary Table 4 Raw variants detected by Lofreq in a rice TILLING population.

Supplementary Table 5 General summary of types of mutations detected in a rice TILLING population.

Supplementary File 1 Details of parameters used to run each of the variant calling tools compared in this work.

## References

[B1] Bajaj D. Srivastava R. Nath M. Tripathi S. Bharadwaj C. Upadhyaya H. D. (2016). EcoTILLING-based association mapping efficiently delineates functionally relevant natural allelic variants of candidate genes governing agronomic traits in chickpea. *Front. Plant Sci.* 7:450. 10.3389/fpls.2016.00450 27148286 PMC4835497

[B2] Bansal V. (2010). A statistical method for the detection of variants from next-generation resequencing of DNA pools. *Bioinformatics* 26 i318–i324. 10.1093/bioinformatics/btq214 20529923 PMC2881398

[B3] Chen L. Hao L. Parry M. A. Phillips A. L. Hu Y. G. (2014). Progress in TILLING as a tool for functional genomics and improvement of crops. *J. Integr. Plant Biol.* 56 425–443. 10.1111/jipb.12192 24618006

[B4] Dashnow H. Bell K. M. Stark Z. Tan T. Y. White S. M. Oshlack A. (2019). *Pooled-parent Exome Sequencing to Prioritise De Novo Variants in Genetic Disease. BioRxiv [Preprint].* Available online at: https://www.biorxiv.org/content/10.1101/601740v1.abstract (Accessed October 28, 2020)

[B5] Duitama J. Kafuri L. Tello D. Leiva A. M. Hofinger B. Datta S. (2017). Deep assessment of genomic diversity in cassava for herbicide tolerance and starch biosynthesis. *Comput. Struct. Biotechnol. J.* 15 185–194. 10.1016/j.csbj.2017.01.002 28179981 PMC5295625

[B6] Garrison E. Marth G. (2012). *Haplotype-based Variant Detection from Short-read Sequencing. arXiv [Preprint].* Available online at: https://arxiv.org/abs/1207.3907 (Accessed October 28, 2020).

[B7] Geourjon C. Deleage G. (1995). SOPMA: significant improvements in protein secondary structure prediction by consensus prediction from multiple alignments. *Bioinformatics* 11 681–684. 10.1093/bioinformatics/11.6.681 8808585

[B8] Henry I. M. Nagalakshmi U. Lieberman M. C. Ngo K. J. Krasileva K. V. Vasquez-Gross H. (2014). Efficient genome-wide detection and cataloging of EMS-induced mutations using exome capture and next-generation sequencing. *Plant Cell* 26 1382–1397. 10.1105/tpc.113.121590 24728647 PMC4036560

[B9] Holme I. B. Gregersen P. L. Brinch-Pedersen H. (2019). Induced genetic variation in crop plants by random or targeted mutagenesis: convergence and differences. *Front. Plant Sci.* 10:1468. 10.3389/fpls.2019.01468 31803209 PMC6868598

[B10] Huang H. W. Mullikin J. C. Hansen N. F., and NISC Comparative Sequencing Program (2015). Evaluation of variant detection software for pooled next-generation sequence data. *BMC Bioinform.* 16:235. 10.1186/s12859-015-0624-y 26220471 PMC4518579

[B11] Irshad A. Guo H. Zhang S. Liu L. (2020). TILLING in cereal crops for allele expansion and mutation detection by using modern sequencing technologies. *Agron. J.* 10:405 10.3390/agronomy10030405

[B12] Kim S. Tai T. H. (2014). Identification of novel rice low phytic acid mutations via TILLING by sequencing. *Mol. Breed.* 34 1717–1729. 10.1007/s11032-014-0127-y

[B13] Kurowska M. Daszkowska-Golec A. Gruszka D. Marzec M. Szurman M. Szarejko I. (2011). TILLING-a shortcut in functional genomics. *J. Appl. Genet.* 52:371. 10.1007/s13353-011-0061-1 21912935 PMC3189332

[B14] Lange V. Böhme I. Hofmann J. Lang K. Sauter J. Schöne B. (2014). Cost-efficient high-throughput HLA typing by MiSeq amplicon sequencing. *BMC Genom.* 15:63. 10.1186/1471-2164-15-63 24460756 PMC3909933

[B15] Ma X. Sajjad M. Wang J. Yang W. Sun J. Li X. (2017). Diversity, distribution of Puroindoline genes and their effect on kernel hardness in a diverse panel of Chinese wheat germplasm. *BMC Plant Biol.* 17:158. 10.1186/s12870-017-1101-8 28931378 PMC5607584

[B16] Manimekalai R. Suresh G. Govinda Kurup H. Athiappan S. Kandalam M. (2020). Role of NGS and SNP genotyping methods in sugarcane improvement programs. *Crit. Rev. Biotechnol.* 40 865–880. 32508157 10.1080/07388551.2020.1765730

[B17] McCallum C. M. Comai L. Greene E. A. Henikoff S. (2000). Targeting induced local lesions in genomes (TILLING) for plant functional genomics. *Plant Physiol.* 123 439–442. 10.1104/pp.123.2.439 10859174 PMC1539256

[B18] McKenna A. Hanna M. Banks E. Sivachenko A. Cibulskis K. Kernytsky A. (2010). The genome analysis toolkit: a mapreduce framework for analyzing next-generation dna sequencing data. *Genome Res.* 20 1297–1303. 10.1101/gr.107524.110 20644199 PMC2928508

[B19] Milburn D. Laskowski R. A. Thornton J. M. (1998). Sequences annotated by structure: a tool to facilitate the use of structural information in sequence analysis. *Protein Eng.* 11 855–859. 10.1093/protein/11.10.855 9862203

[B20] Missirian V. Comai L. Filkov V. (2011). Statistical mutation calling from sequenced overlapping DNA pools in TILLING experiments. *BMC Bioinform.* 12:287. 10.1186/1471-2105-12-287 21756356 PMC3150297

[B21] Perera I. Fukushima A. Akabane T. Horiguchi G. Seneweera S. Hirotsu N. (2019). Expression regulation of myo-inositol 3-phosphate synthase 1 (INO1) in determination of phytic acid accumulation in rice grain. *Sci. Rep.* 9:14866. 10.1038/s41598-019-51485-2 31619750 PMC6795888

[B22] Raja R. B. Agasimani S. Jaiswal S. Thiruvengadam V. Sabariappan R. Chibbar R. N. (2017). EcoTILLING by sequencing reveals polymorphisms in genes encoding starch synthases that are associated with low glycemic response in rice. *BMC Plant Biol.* 17:13. 10.1186/s12870-016-0968-0 28088172 PMC5423428

[B23] Rubinstein M. Eshed R. Rozen A. Zviran T. Kuhn D. N. Irihimovitch V. (2019). Genetic diversity of avocado (Persea americana Mill.) germplasm using pooled sequencing. *BMC Genom.* 20:379. 10.1186/s12864-019-5672-7 31092188 PMC6521498

[B24] Slota M. Maluszynski M. Szarejko I. (2017). “Bioinformatics-based assessment of the relevance of candidate genes for mutation discovery,” in *Biotechnologies for Plant Mutation Breeding*, eds Jankowicz-Cieslak J. Tai T. H. Kumlehn J. Till B. J. (Cham: Springer Nature), 263–280.

[B25] Taylor N. E. Greene E. A. (2003). PARSESNP: a tool for the analysis of nucleotide polymorphisms. *Nucleic Acids Res.* 31 3808–3811. 10.1093/nar/gkg574 12824424 PMC168980

[B26] Till B. J. Cooper J. Tai T. H. Colowit P. Greene E. A. Henikoff S. (2007). Discovery of chemically induced mutations in rice by TILLING. *BMC Plant Biol.* 7:19. 10.1186/1471-2229-7-19 17428339 PMC1858691

[B27] Tsai H. Howell T. Nitcher R. Missirian V. Watson B. Ngo K. J. (2011). Discovery of rare mutations in populations: TILLING by sequencing. *Plant Physiol.* 156 1257–1268. 10.1104/pp.110.169748 21531898 PMC3135940

[B28] Wang T. L. Uauy C. Robson F. Till B. (2012). TILLING in extremis. *Plant Biotechnol. J.* 10 761–772. 10.1111/j.1467-7652.2012.00708.x 22651686

[B29] Wei Z. Wang W. Hu P. Lyon G. J. Hakonarson H. (2011). SNVer: a statistical tool for variant calling in analysis of pooled or individual next-generation sequencing data. *Nucleic Acids Res.* 39:132. 10.1093/nar/gkr599 21813454 PMC3201884

[B30] Wilm A. Aw P. P. K. Bertrand D. Yeo G. H. T. Ong S. H. Wong C. H. (2012). LoFreq: a sequence-quality aware, ultra-sensitive variant caller for uncovering cell-population heterogeneity from high-throughput sequencing datasets. *Nucleic Acids Res.* 40 11189–11201. 10.1093/nar/gks918 23066108 PMC3526318

[B31] Yu S. Liao F. Wang F. Wen W. Li J. Mei H. (2012). Identification of rice transcription factors associated with drought tolerance using the ecotilling method. *PLoS One* 7:e30765. 10.1371/journal.pone.0030765 22348023 PMC3278407

