# Supplementary material for: Accurate, Efficient and User-Friendly Mutation Calling and Sample Identification for TILLING Experiments
Source: Front Genet. 2021 Feb 3;12:624513. doi: 10.3389/fgene.2021.624513 (PMC7886796; doi:10.3389/fgene.2021.624513)
Supplement: Supplementary File 1 — Details of parameters used to run each of the variant calling tools compared in this work. [file Data_Sheet_1.PDF]

CRISP

Command:
path/to/CRISP --bams bam_files_paths.txt --ref path/to/Reference.fa --VCF out.vcf --useduplicates 1 --qvoffset 33 >& out.log

Parameters:
--useduplicates 1
--qvoffset 33
Ploidy of each pool was included as a separate column in the file "bam_files_paths.txt" with the option PS="ploidy".

freebayes

Command:
path/to/freebayes -f path/to/Reference.fa --ploidy 96 --pooled-discrete --min-mapping-quality 20 in.bam --vcf out.vcf >& out.log

Parameters:
--ploidy 96 (according to pool 96 or 72)
--pooled-discrete
--min-mapping-quality 20

GATK

Command:
path/to/gatk --java-options "-Xmx3500m" HaplotypeCaller -R path/to/Reference.fa -I in.bam -O out.vcf --heterozygosity 0.5 -ploidy 96 --max-reads-per-alignment-start 0 >& out.log

Parameters:
--heterozygosity 0.5
-ploidy 96 (according to pool 96 or 72)
--max-reads-per-alignment-start 0

lofreq

Command:
lofreq call --no-default-filter -m 20 -f path/to/Reference.fa -o out.vcf in.bam >& out.log

Parameters:
--no-default-filter
-m 20

NGSEP simulations

Command:
java -Xmx3500m -jar path/to/NGSEPcore_4.1.0.jar SingleSampleVariantsDetector -i in.bam -r path/to/Reference.fa -o out.vcf -sampleId poolID -h 0.5 -ploidy 96 -maxAlnsPerStartPos 0 -maxBaseQS 100 -psp >& out.log

Parameters:
-h 0.5
-ploidy 96 (according to pool 96 or 72)
-maxAlnsPerStartPos 0
-maxBaseQS 100
-psp

NGSEP real data

Command:
java -Xmx3500m -jar path/to/NGSEPcore_4.1.0.jar SingleSampleVariantsDetector -i in.bam -r path/to/Reference.fa -o out.vcf -sampleId poolID -h 0.5 -ploidy 96 -maxAlnsPerStartPos 0 -maxBaseQS 30 -psp >& out.log

Parameters:
-h 0.5
-ploidy 96 (according to pool 96 or 72)
-maxAlnsPerStartPos 0
-maxBaseQS 30
-psp

SNVer

java -Xmx3500m -jar path/to/SNVerPool.jar -i bams_folder/ -r path/to/Reference.fa -c poolInfo.txt -o out_prefix >& out.log

Parameters are determined in the file poolInfo.txt, which has to include the name of the files, number of haploids and number of samples per pool, mapping quality (mq 20) and base quality (bq 17).
